# Mechanocatalytic Hydrogen Generation in Centrosymmetric Barium Dititanate

**DOI:** 10.1002/advs.202404483

**Published:** 2024-08-09

**Authors:** Yumeng Du, Wei Sun, Xiaoning Li, Chongyan Hao, Jianli Wang, Yameng Fan, Jincymol Joseph, Changhong Yang, Qinfen Gu, Yun Liu, Shujun Zhang, Zhenxiang Cheng

**Affiliations:** ^1^ Institute for Superconducting and Electronics Materials Faculty of Engineering and Information Science University of Wollongong Squires Way North Wollongong NSW 2500 Australia; ^2^ Shandong Provincial Key Laboratory of Preparation and Measurement of Building Materials University of Jinan Jinan 250022 China; ^3^ Center for neutron scattering and advanced light sources Dongguan University of Technology Dongguan 52300 China; ^4^ Australia Synchrotron (ANSTO) 800 Blackburn Rd Clayton VIC 3168 Australia; ^5^ Research School of Chemistry Australian National University Canberra ACT 2601 Australia

**Keywords:** barium dititanate, dipole layers, flexocatalysis, hydrogen generation, mechanocatalysis

## Abstract

Novel phase of nano materials that break the traditional structural constraints are highly desirable, particularly in the field of mechanocatalysis, offering versatile applications ranging from energy to medical diagnosis and treatment. In this work, a distinct layered barium dititanate (BaTi_2_O_5_) nanocrystals using a pH‐modulated hydrothermal method is successfully synthesized. These nanocrystals exhibit outstanding hydrogen generation capability (1160 µmol g^−1^ h^−1^ in pure water) and demonstrate remarkable performance in organic dye degradation using ultrasonication. The crystal structure of this newly discovered BaTi_2_O_5_ phase, is determined by a combination of synchrotron Powder Diffraction refinement and X‐ray adsorption techniques, including X‐ray Absorption Near Edge Structure (XANES) and Extended X‐ray Absorption Fine Structure (EXAFS). Density Functional Theory calculations revealed that the newly‐discovered BaTi_2_O_5_ phase demonstrates dipole moments along the z‐axis, distributed in an antiparallel direction within a single unit cell. These inherent dipoles induce a surface polarization and a ferroelectric‐flexoelectric response under mechanical stimuli when the materials go to nano dimension. With a band alignment well‐suitable for hydrogen and reactive oxygen species generation, this BaTi_2_O_5_ phase demonstrates promising potential for Mechanocatalysis. The discovery of this distinct phase not only enriches the material candidates for mechanocatalysis but also provides valuable insights.

## Graphical abstract

A novel insight of energy conversion mechanism in mechanocatalysis is delved by uncovering a new structural catalyst, centrosymmetric BaTi_2_O_5_, displaying two anti‐directional dipoles in a single unit cell with 26.1 µC cm^−^^2^ per dipole layer, suggesting its potential dual role as an effective flexo‐catalyst and a quasi‐ferro‐catalyst, exhibiting different dipole layer conditions (odd‐layer and even‐layer) subjected to inhomogeneous stress.

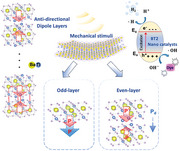

## 1 Introduction

Mechanocatalysis refers to a catalytic process that applies mechanical forces to activate or accelerate chemical reactions, such as through grinding, milling, or ultrasound.^[^ ^1^ ^]^ Unlike traditional catalysis, which relies on chemical reagents, mechanocatalysis offers unique advantages in terms of energy efficiency and selectivity, contributing to more sustainable and environmentally friendly chemical reactions, particularly for applications under special circumstances. While mechanocatalysis holds great potentials for applications in green chemistry, medical treatment, and diagnosis, there are challenges that need to be addressed to enable large‐scale applications.^[^ ^2^ ^]^

The primary challenge in mechanochemistry lies in the limitation of stress‐responsive functional materials. This limitation arises from the requirement for a non‐centrosymmetric crystal structure, specifically associated with piezoelectric materials, in order to convert mechanical energy to electrical energy.^[^ ^3^ ^]^ To address this limitation, the concept of flexocatalysis has been developed and explored, leveraging the universality of flexoelectricity that relies on the strain gradient induced energy conversion scheme, exhibiting in all dielectrics.^[^ ^4^ ^]^ Unlike piezocatalysis, which relies on the piezoelectric effect to generate electrical potential and trigger chemical reactions when subjected to external mechanical force, flexocatalysis originates from charges induced by inhomogeneous strain. In other words, the material can become polarized due to the existence of a strain gradient.^[^ ^5^ ^]^ Nevertheless, regarding real‐world applications, appreciable flexoelectricity is constrained by particle sizes as well as material permittivity, which necessitates more comprehensive studies to validate and support these emerging strategies in mechanocatalysis.^[^ ^6^ ^]^ Similarly, recent research has directed attention toward the investigation of surface piezoelectricity as an alternative strategy to broaden catalysts selection.^[^ ^3^ ^]^ Surface piezoresponse can originate from the breaking of local symmetry, such as defects or crystal partial distortions, introducing dipoles in specific areas that may be adjusted by external mechanical stresses.^[^ ^7^ ^]^ Hence, this approach has the potential to eliminate the structural constraints in material selection.

These advancements underscore a shift in previously proposed strategies, aiming to enrich mechanocatalysis with diverse mechanisms, opens up more opportunities for future applications of mechanical energy‐based chemical reactions, incorporating a wide‐range of feasible catalysts.

In this work, we delve into a novel insight of energy conversion mechanism in mechanocatalysis by uncovering a new structural catalyst, layered barium dititanate (BaTi_2_O_5_(BT2)). This distinctive structure of BT2 was synthesized using a pH‐modulated hydrothermal method, displaying two anti‐directional dipoles in a single unit cell. This unique characteristic suggests its potential dual role as an effective flexo‐catalyst and a quasi‐ferro‐catalyst, exhibiting different dipole layer conditions subjected to inhomogeneous stress. The mechanocatalytic generation of free radicals using ultrasonication has been investigated for hydrogen generation and organic dye degradation, demonstrating outstanding performance rivaling its well‐known ferroelectric counterpart, BaTiO_3_.^[^ ^8^ ^]^ Structural details of this new BT2 are characterized by Synchrotron power diffraction and X‐ray adsorption spectroscopy, with a confirmed polarization of 26.1 µC cm^−2^ derived from half unit cell layer, as affirmed by Density Functional Theory (DFT) theoretical calculation. This work identifies a new functional material for use in mechanocatalysis. Its unique crystal structure and physical properties not only provide fresh insights for the development of catalytic mechanisms, but also inspire multidisciplinary explorations.

## 2 Results and Discussion

### 2.1 Crystal Structure and Morphology

**Figure 1:**
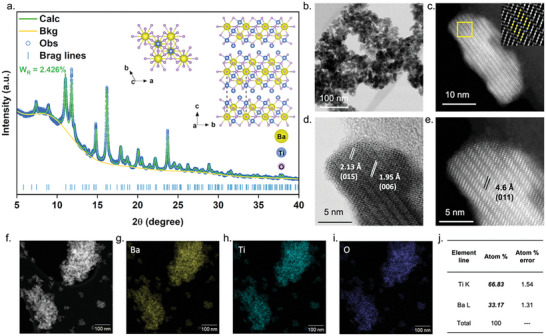
Crystal structural characterization and morphology. a) Synchrotron powder diffraction patterns of BT2 nanoparticles and Rietveld refinement results with crystalline structure demonstration on the right top conner. A single unit cell is outlined by the dashed rectangle. (WR: data residuals) b) Scanning transmission electron microscopy bright field (STEM BF) images of BT2 nanoparticles. c) High‐angle annular dark field (STEM HAADF) image of the edge‐on particles exactly alone z‐axis. The inset is the inverse fast Fourier transform (IFFT) image of the selected area in yellow color, corresponding to the overlapped atomic structure (colored) refined from the synchrotron powder diffraction. d) STEM BF image of BT2 with lattice plane illustrations. e) STEM DF image of BT2 with the lattice plane illustration. f–i) STEM EDS images of BT2 nanoparticles. j) Atomic distribution of BT2 nanoparticles.

BT2 nanopowders have been synthesized by pH‐modulated hydrothermal methods, in which uniform precursors can be achieved through the precipitation process of adding ionic metal sources into a buffer solution with a pH value ≈ 10.6. The BT2 nanopowders achieved in this work represent a newly‐discovered phase of bismuth dititanate, distinguished from the previously documented phase in an orthorhombic structure with space group *Pnma*.^[^ ^9^ ^]^ **Figure** **1**a shows the synchrotron powder diffraction (SPD) pattern of this new phase BT2 along with its crystal structure refined using Rietveld analysis. This crystal structure belongs to the trigonal space group *P −3m1*, with lattice parameters a = 5.7155, b = 5.7155, and c = 11.6490 Å. The detailed unit cell demonstration and structural parameters can be found in Figure S1 and Table S1 (Supporting Information). The basic unit cell (Ba_3_Ti_6_O_15_), outlined by a dashed line in the top‐left corner of Figure 1a, contains three formular units (BaTi_2_O_5_), presenting a double‐layer structure with Ti‐O_(6)_ heptahedra located at interlayer.

The morphology of BT2 nanopowders has been unveiled by STEM images illustrated in Figure 1b. The average particle size is ≈10 to 20 nm with an irregular shape. The IFFT image, positioned in the top‐left corner of Figure 1c, displays a selected area of the STEM image captured along the crystal axis, revealing a clear atomic distribution of Ba (brightest) and Ti (less bright). Notably, the atomic arrangement derived from the SPD refinement (colored atoms) can perfectly align with the actual atomic distribution, demonstrating the precise structural prediction of this new phase BT2. The crystalline structure of BT2, with d‐spacing of 2.13 and 1.95 Å, corresponds to the lattice planes of (015) and (006), as shown in Figure 1d. The d‐spacing of 4.6 Å in Figure 1e corresponds to the (011) lattice plane. Further details regarding to the morphology and lattice planes of these new BT2 nanoparticles are presented in Figure S2 and Table S2 (Supporting Information). In addition, the STEM EDS images displayed in Figure 1f–i illustrate the uniform elemental distribution of Ba, Ti, and O, with a quantitative elemental Ba/Ti ratio of 2:1 as shown in Figure 1j, further substantiated by XPS survey results presented in Figure S3 (Supporting Information).

**Figure 2:**
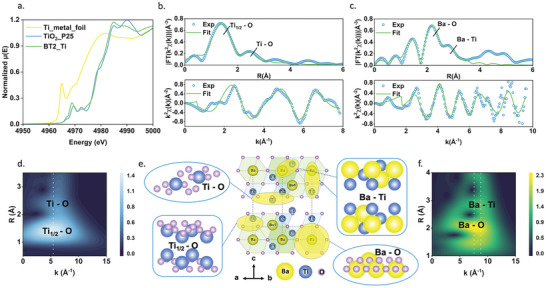
Structural details analysis by synchrotron X‐ray absorption spectra. a) The Ti K‐edge X‐ray adsorption near‐edge structure (XANES) spectra of the BT2 sample, TiO_2_ (P25), and Ti metal foil, respectively. b) The Ti K‐edge Extended X‐ray adsorption fine structure (EXAFS) spectra with fitting results. c) The Ba L‐edge EXAFS spectra with fitting results. d) Wavelet transformations (WTs) of k2‐weighted 𝜒(k) Ti K‐edge EXAFS data. e) Atomic structural illustration of information conveyed from WTs images using refinement unit cell from synchrotron powder diffraction (SPD) f) Wavelet transformations (WTs) of k2‐weighted 𝜒(k) Ba L‐edge EXAFS data.

X‐ray absorption spectroscopy including X‐ray absorption near‐edge structure (XANES) and extended X‐ray absorption fine structure (EXAFS) has been employed to elucidate the valence states and local atomic structure of the new phase BT2, further substantiating the preceding structural analysis. In **Figure** **2**a, Ti K‐edge XANES spectra for Ti metal foil, TiO_2_ (P25), and the BT2 sample are displayed, with adsorption edge positions indicating Ti^4+^ in BT2 sample. As shown in Figure 2b,c, quantitative EXAFS fitting has been conducted to obtain structural parameters and determine the precise atomic coordination configuration of Ti and Ba atoms for BT2. The details of the fitting parameters have been displayed in **Table** **1**, indicating the high precision of the refined structure. The Fourier transform (FT) k_2_‐weighted Ti K‐edge EXAFS spectrum in Figure 2b illustrates two high‐intensity peaks before and after 2 Å, corresponding to Ti_1/2_‐O in Ti‐O_(6)_ octahedra and Ti‐O in the interlayers located between the two half unit cells. Peaks labeled in FT k_2_‐weighted Ba L‐edge EXAFS spectrum in Figure 2c, correspond to Ba‐O and Ba‐Ti interactions in the BT2 structure, respectively. These results can be virtualized through the EXAFS wavelet transformations (WTs) plots (Figure 2d,f), in which the intensity maxima correspond to the highest peaks in FT spectra. For example, in Figure 2d, the brightest peak indicates the Ti_1/2_‐O interaction at 1.5 Å, while the second brightest peak represents the Ti‐O interaction at 2.5 Å, and these two peaks align at around k = 5.5 Å^−1^, suggesting that the absorbing atom (Ti) interacts with the same atom (O). However, in Figure 2f, the second brightest peak at 2.9 Å has a wavenumber shift at around k = 9 Å^−1^ in comparison to the brightest peak at k = 7.5 Å^−1^, indicating a different neighboring interaction (Ba‐Ti) in contrast to Ba‐O. The structural fingerprint uncovered by XAS analysis is consistent with the refined crystal structure obtained from SPD, and the identified bonds have been demonstrated in Figure 2e.

**Table 1:** BT2 sample EXAFS fitting results.

Coordination Type	N	R/Reff [Å]	σ2 [× 10^−3^Å2]	∆E[eV]	R‐factor
Ti_1/2_‐O	3	1.90760/1.90290	1.15	3.367	0.017
Ti‐O (layered)	6	2.90489/2.91230	9.00	−8.885	0.018
Ba‐O_path1	6	2.72260/2.78860	3.00	11.530	0.006
Ba‐O_path2	6	2.95210/2.86870	4.00	0.875	0.006
Ba‐Ti	6	3.37640/3.48640	9.00	1.200	0.006

N: Coordination number of the refined atom. R: Refined atomic distance. R_eff_: Reference atomic distance derived from SPD. σ^2^: Debye‐Waller factor. ∆E: Energy shift. R‐factor: Fitting precision.

### 2.2 Catalytic Performance

**Figure 3:**
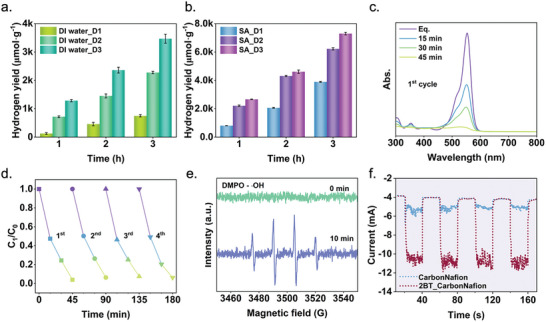
Catalytic performance. a) Hydrogen evolution yield of BT2 catalysts in pure water under ultrasonic irradiation. D1, D2, and D3 represent for the catalytic procedure conducted over three consecutive days using the same catalysts. The error bar is determined by the maximum difference observed among multiple trials conducted under the same experimental conditions. b) Hydrogen evolution yield of BT2 catalysts in 0.05 M Na_2_SO_3_ as SA using ultrasonication. c) Adsorption spectra of RhB dye degradation using BT2 nanoparticles. d) Rhodamine B (RhB) dye degradation stability test plotted in decrease of dye concentration. (C_0_: original RhB dye concentration, and C_1_: degraded RhB concentration after catalysis with different time intervals) (e) Hydroxyl radicals’ detection by electron paramagnetic resonance (EPR) spectroscopy. f) The current response of BT2 catalysts by ultrasonication tested in the electrochemical station. Carbon paper served as the reference data.

**Figure** **3** depicts the catalytic performance of BT2 nanoparticles by ultrasonication including hydrogen generation and organic dye degradation. Hydrogen generation experiments were conducted in both pure water (Figure 3a) and in the presence of sacrificial agent (SA), (Figure 3b) in darkness, avoiding potential synergistic catalysis from photoactive effects, despite uncertainty about the sensitivity of BT2 to light. The role of SA in the catalysis process is to act as a hole scavenger, effectively reducing charge recombination and ultimately enhancing hydrogen production.^[^ ^10^ ^]^ Hydrogen traces were monitored hourly over 3‐h period each day up to three days. It has been discovered that the catalytic activity of BT2 improves withing an increasing immersion time. In pure water, the average hydrogen generation yield increased from 249.5 µmol g^−1^h^−1^ on day 1, to 758.2 µmol g^−1^h^−1^ on day 2 and finally to 1156.3 µmol g^−1^h^−1^ on day 3, and a similar trend is observed in the case of SA. Speculation arises that the high surface hydrophilicity and moisture absorption capability of newly synthesized layered BT2 nanoparticles via the hydrothermal method may allow water molecules to penetrate bonded layers, potentially forming new chemical bonds during immersion. Under ultrasound stimuli, this process could involve the chemical or physical adsorption of polar molecules, such as hydroxyl species in water, which could alter the original chemical bonds and interface area of BT2, thereby potentially influencing the electrical properties of the catalyst.^[^ ^11^ ^]^

Furthermore, organic RhB dye degradation was conducted to investigate the potential generation of reactive oxygen species (ROS) by BT2. As demonstrated in Figure 3c,d, BT2 successfully degrades RhB dye (>96%) within 45 min, using a catalyst dosage of 2 mg/10 mL, and catalyst stability can be maintained for at least 4 cycles. In the recycling procedure, the catalysts were centrifuged and dried for subsequent use without further immersion. Hence, the performance in organic dye degradation did not exhibit a significant difference compared to hydrogen generation. EPR results confirm the presence of ROS generated by BT2 with the peak signal in Figure 3e indicating the presence of hydroxyl radicals.

The current response of BT2 catalysts under ultrasonic irradiation was detected using an electrochemical station with a three‐electrode cell.^[^ ^5a^ ^]^ In the test, BT2 nanopowders and Nafion mixture were deposited on a piece of carbon paper and fully immersed in the electrolyte. In Figure 3f, when ultrasonic power is applied, the current response of BT2 sample shows a significantly more pronounced increase (from ≈−5 mA to ≈−11 mA) compared to the case of the reference carbon paper. This supports the active mechano‐response of BT2 nanoparticles under ultrasonic irradiation.

Figures S4 and S5 (Supporting Information) demonstrate the Synchrotron powder diffraction (SPD) patterns and X‐ray photoelectron spectroscopy (XPS) results of BT2 sample before and after catalysis, aiming to investigate the structural and surface stability of the catalysts. Apparently, both the position and intensity of the SPD peaks of the used BT2 powders remain identical, revealing robust stability after catalysis. Observed XPS signal O 1s in Figure S5 (Supporting Information) reveals an increase in the peak area of surface moistures after catalysis, indicating a minor surface modification that may attributed to the polar molecules attraction in water after prolonged vibration and immersion. This observation may provide insight into the earlier speculation regarding surface moisture absorption during immersion, as mentioned in the performance section.

### 2.3 Catalytic Mechanisms Demonstration

**Figure 4:**
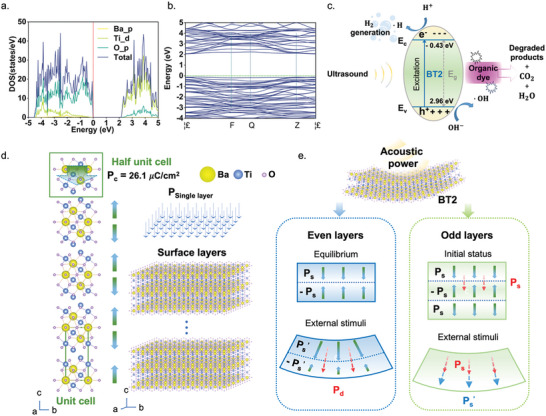
Catalytic mechanisms. a) Theoretical calculation of density of state (DOS) for BT2 sample. The Fermi level is indicated by the vertical dot line and set to zero. b) Theoretical calculation of electronic energy band structure. The Fermi level is indicated by horizontal dot line and set to zero. c) Catalytic processes illustration with catalyst energy band diagram estimated from experiment data. d) Theoretical calculation of single‐layer polarization combined with near‐surface structural demonstration. e) Catalytic mechanisms inference in two possible scenarios. (P_s_: polarization in a single layer with the same dipole direction. −P_s_: polarization with anti‐direction of P_s_. The layers with anti‐directional polarizations of P_s_ and −P_s_ are neighboring layers. $P_{s}^{\prime }$ and ${-P}_{s}^{\prime }$: polarizations under external stimuli. P_d_: the net polarization under external stimuli (the difference between $P_{s}^{\prime }$ and ${-P}_{s}^{\prime }$).).

The atomic orbital projected density of states for the new structure BT2 and its electronic band structure have been calculated using first‐principles within Density Functional Theory with consideration of spin‐orbit coupling to enhance the comprehension of catalytic mechanisms. As shown in **Figure** **4**a, the calculated bandgap Eg is ≈2.18 eV, which is lower than the experimental value of 3.39 eV, due to the underestimation of bandgap by DFT calculation. The total density of states (DOS) of BT2 is significantly influenced by O 2p and Ti 3d orbitals, with O 2p making the strongest contributions to the valence band while Ti 3d playing a predominant role in the conduction band. Furthermore, it is noteworthy that three flat bands are evident in the electronic band structure shown in Figure 4b. These correspond to the first two DOS peaks in the conduction band, occurring at ≈2.3 and ≈2.6 eV, attributed to Ti 3d orbitals, as well as the top valence band attributed to O 2p orbitals corresponding to the ≈−0.1 eV DOS peak. Aside from the theoretical calculations, optical spectroscopy, including UV–vis spectroscopy and UPS, has been employed to estimate the BT2 band structure. Figure S6a,b (Supporting Information) provides details of the estimated energy diagram of BT2, featuring a bandgap value of 3.39 eV, a conduction band at −4.01 eV (−0.43 eV vs NHE), and a valence band at −7.40 eV (2.96 eV vs NHE) relative to the vacuum level. Eligible catalytic processes, including hydrogen generation and organic dye degradation, have been demonstrated in Figure 4c, and align with the estimated energy band levels. The notable difference in bandgap values between theoretical and experimental estimations may be attributed to the presence of flat bands, which arise from strongly correlated electrons, challenging conventional band theory.^[^ ^12^ ^]^ In addition, an polarization value of 26.1 µC m^−^^2^ distributed across layered BT2 along the c‐axis has been attained by integrating the charge density of the halve of the unit cell. As illustrated in Figure 4d, the polarization is distributed anti‐directionally within the crystal structure with one direction in one dipole layer and the opposite direction in the neighboring dipole layer. However, in a single unit cell, the crystal remains neutrally polarized due to the presence of two anti‐directional dipoles.

Hence, combining all the identified features of this new BT2, we can deduce the contributing mechanisms behind the aforementioned effective mechanocatalysis performance. The distinctive BT2 nanoparticles, consisting of multiple dipole layers, prompt consideration of energy conversion processes on the catalyst surface in two scenarios: the even dipole layers scenario and the odd dipole layers scenario. As shown in Figure 4e, taking the bi‐dipole‐layer condition for example, under an inhomogeneous external mechanical stimulus – in this case, the acoustic power from bubble cavitation, the equilibrium magnitudes of polarizations P_s_ and −P_s_ are no longer the same due to the presence of flexoelectric polarization resulting from the strain gradient and local symmetry broken. The direction of flexoelectric polarization generated aligns with the strain gradient vector. Consequently, the oriented flexoelectric polarization may reinforce one anti‐directional (P_s_) while attenuating the other, resulting in a net polarization (P_d_) as depicted by the “External stimuli” in Figure 4e. The presence of dynamic net polarization induced by dynamic external stimuli such as ultrasound, can activate and accelerate charges to the catalysts’ surface, facilitating electron transfer and initiating redox reactions.

The following equations show the potential pathway of redox reaction for hydrogen generation (H·: hydrogen free radicals) and organic degradation (·OH: hydroxyl free radicals) as depicted in Figure 4c:

(1) $$H^{+}+e^{-}=H\cdot$$

(2) $$H\cdot +H\cdot =H_{2}$$

(3) $$OH^{-}+h^{+}=\cdot \mathrm{OH}$$

(4) $$\mathrm{Organicdye}+\cdot \mathrm{OH}=\mathrm{Intermediates}+CO_{2}+H_{2}O$$

In an odd dipole layer scenario, contrary to even layers, the initial status is no longer net polarization‐neutral due to the presence of the surface odd layer. It's important to note that this is a rare occurrence, as the particle surface is more likely to terminate at the interlayer position due to weak bonding. In this scenario, the catalytic process can be explained as quasi‐ferrocatalysis, due to the presence of build‐in polarization (P_s_ in red), akin to the spontaneous polarization in ferroelectric materials. This net build‐in polarization of the nanoparticle can be dynamically adjusted by external mechanical stimuli, generating an electrical potential for charge movement, thereby triggering specific redox reactions. Hence, in either scenario, the catalysis becomes possible, contributing to the remarkable and stable catalytic performance of BT2. Additionally, the flat bands depicted in the electronic band structure may also influence the catalytic activity, as flat bands can ensure a large electron density at specific energy levels.

## 3 Conclusion

This study identified a groundbreaking functional catalyst, a distinct phase barium dititanate characterized by centrosymmetric trigonal crystal structure resolved through a combination of multiple approaches including STEM, Synchrotron powder diffraction, and X‐ray absorption spectroscopy (XANES and EXAFS). The crystal features two anti‐directional dipoles in a single unit cell, exhibiting a polarization of up to 26.1 µC cm^−^^2^ within one dipole layer, as confirmed by DFT calculations. This nano‐sized BT2 catalyst demonstrates remarkable catalytic efficiency in hydrogen generation and organic dye degradation using ultrasonication. The two proposed catalytic mechanisms in distinct scenarios, that is, quasi‐ferroelectric flexoelectric response, underscore the outstanding catalytic performance of BT2. The electronic band structure simulations reveal the presence of flat bands, suggesting their potential influence on catalytic capabilities due to the likelihood of high electron density within corresponding energy levels. This work not only advances the field of mechanocatalysis with innovative structural catalysts based on new mechanisms but also contributes valuable insights that could inspire future explorations in material science and condensed matter physics.

## Conflict of Interest

The authors declare no conflict of interest.

## Supporting information

Supporting Information

## Data Availability

The data that support the findings of this study are available from the corresponding author upon reasonable request.
