# Supplementary material for: Mechanocatalytic Hydrogen Generation in Centrosymmetric Barium Dititanate
Source: Adv Sci (Weinh). 2024 Aug 9;11(38):2404483. doi: 10.1002/advs.202404483 (PMC11481254; doi:10.1002/advs.202404483)
Supplement: Supplementary file 1 — Supporting Information [file ADVS-11-2404483-s001.docx]

**Supplementary information**

**Mechanocatalytic Hydrogen Generation in Centrosymmetric Barium Dititanate**

Yumeng Du,^[a]^ Wei Sun,^[b]^ Xiaoning Li,^[a]^ Chongyan Hao,^[a]^ Jianli Wang,^[a]^ Yameng Fan,^[a]^ Jincymol Joseph,^[a]^ Changhong Yang,^[b]^ Qinfen Gu,^[c]^ Yun Liu,^[d]^ Shujun Zhang,^*[a]^ and Zhenxiang Cheng^*[a]^

^a^Institute for Superconducting and Electronics Materials, Faculty of Engineering and Information Science, University of Wollongong, Squires Way, North Wollongong, 2500, Australia

^b^Shandong Provincial Key Laboratory of Preparation and Measurement of Building Materials, University of Jinan, Jinan 250022, China

^c^Australian Synchrotron (ANSTO), 800 Blackburn Rd Clayton, VIC 3168, Australia

^d^Research School of Chemistry, Australian National University, 2601 Canberra, Australia
E-mail: shujun@uow.edu.au, cheng@uow.edu.au

Experimental Methods

1. Hydrothermal synthesis of BaTi_2_O_5_ (BT2) nanopowders

5 mmol titanium butoxide (97 %) was dispersed in 5 mL 1 M HNO_3_ with magnetic stirring until complete dispersion was achieved. 20 mL distilled water was then added to the acid solution with half an hour magnetic stirring labelled as solution 1. Additionally, 10 mmol Ba(NO_3_)_2_ (99 %) was dispersed in 25 mL distilled water with magnetic stirring until fully dissolved, resulting in a transparent solution labelled as solution 2. Subsequently, solution 1 and solution 2 were carefully dispensed using a peristaltic pump at a controlled speed of 400 µL/min into a buffer solution with a pH of 10 -11. White precursors were successfully formed during this process and these precursors were then mixed with 30 mL 3M NaOH into a 50 mL Teflon-lined stainless-steel autoclave for hydrothermal treatment (180 °C, 48 h). After autoclave naturally cooling down to room temperature, synthesized sample powders were washed by distilled water and dried at 60 °C overnight.

1. Characterizations for crystal structure and electronic structures

The powder diffraction (PD) patterns, Ba L3-edge and Ti K-edge bulk X-ray adsorption spectroscopy (XAS) spectra (including EXAFS and XANES) were collected from Australian Synchrotron (AS), Australia Nuclear Science and Technology Organization (ANSTO). Athena and Artemis were used for processing the XAS data.^[1]^ The morphology of BT2 nanopowders were examined by scanning transmission electron microscope (STEM, JEM ARM-200F) with a double aberration corrector. Elemental distribution was scanned by STEM using energy dispersive spectroscopy (EDS). Surface valence states were analyzed by X-ray photoelectron spectrometer (XPS, Nexsa Thermo Fisher Scientific), and valence band was estimated from the spectrum collected by an ultraviolet photoelectron spectroscopy (UPS, Thermo Fisher Scientific). Energy band gap (E_g_) was estimated from the diffuse reflectance spectrum (DRS) detected by ultraviolet-visible (UV-Vis) spectroscopy (Shimadzu UV-3600) equipped with an integrated sphere. Adsorption spectra of degraded RhB dye solution were collected by ultraviolet-visible (UV-Vis) spectroscopy (Shimadzu UV-1900). Hydroxyl free radicals were detected by electron paramagnetic resonance (EPR, bruker EMXplus) spectroscopy at ambient temperature, using 5,5-Dimethyl-1-Pyrroline N-Oxide (DMPO) as trapping agent.

1. Hydrogen evolution experiment

Hydrogen evolution experiment was conducted in an ultrasonic bath (200 W, 40 kHz, Powersonic 610, Kleentek Australia) with temperature control. 2 mg BT2 sample was dispersed into either 10 mL distilled water or 10 mL 0.05 M Na_2_SO_3_ (Sacrificial agent). The dispersion was then sealed in a 45 mL borosilicate tube. Prior to sealing, the air inside the tube was purged using argon. During ultrasonic irradiation, the tube containing the sample dispersion was placed in a fixed position within an ultrasonic bath. The water level in the ultrasonic bath was maintained at a constant level. The experiment was conducted in dark surrounding where the water temperature was controlled at room temperature through a circulation system during the test. Hydrogen yield was sampled hourly using 1mL syringe (Hamilton, US) and injected into a gas chromatograph (GC 2600, Shanghai Ruimin, China) with Thermal Conductivity Detector (TCD).

1. Organic dye degradation experiment

10 mg sample powders were dispersed into 50 mL 5 mg/L Rhodamine B (RhB) dye solution with magnetic stirring for 2 hours and kept in darkness overnight to reach adsorption and desorption equilibrium status. The RhB dye degradation experiment was carried out in the same ultrasonic bath used for hydrogen evolution. 10 mL of the mixture, containing an average catalyst dosage of 2 mg per 10 mL, was placed into a borosilicate tube for ultrasonic irradiation at different intervals (15 min, 30 min and 45 min). The position of the tube and the water level in the ultrasonic bath were kept constant throughout the test. Additionally, the water temperature was controlled at room temperature using a water circulation system. Subsequently, the degraded dye solutions were centrifuged for adsorption spectrum acquisition. In the recycling procedure, the catalysts were centrifuged and dried for subsequent use without further immersion.

1. Flexoelectric response detection by electrochemical station using ultrasonication

The transient current signals induced by ultrasonic irradiation were detected through an electrochemical station. The experimental setup involved a three-electrode cell, utilizing Ag/AgCl as the reference electrode. The sample powders mixed with 25 % isopropyl alcohol and nafion were deposited onto a carbon paper and securely held by the working electrode. 0.5 M Na_2_SO_4_ was used as electrolyte and a 1.2 V potential relative to the reference electrode was applied to measure the current piezoresponse. During the measurement process, the catalysts were completely submerged in the electrolyte, and the cell was positioned at a fixed location within the ultrasonic bath. The power of ultrasonic bath was controlled on and off alternately with a 20 s time interval.

1. DFT calculation

The atomic properties and electronic structure of the materials were calculated using first-principles simulations within DFT with consideration of spin-orbit coupling^[2]^. The projected augmented wave pseudopotentials method was used as implemented in the Vienna *Ab* initio Simulation Package (VASP).^[3]^ The exchange correlation energy was calculated using the generalized gradient approximation (GGA) of the Perdew-Burke-Ernzerhof form^[4]^. The plane wave cutoff energy was set to 500 eV. For the calculation of heterostructures, a centered 10 × 10 × 5 Monkhorst-Pack *k*-point mesh was used.^[5]^ Utilizing the conjugate gradient method, the plane lattice constant and atomic coordinates were fully relaxed until the energy and force converge to 10^-5^ eV and 10^–2^ eV/Å, respectively. The electronic self-consistency convergence criterion is set as 10^–8^ eV, in order to accurately calculate polarization. The overall dipole moment parallel to the *z* axis can be calculated by directly integrating the charge density over the half of the unit cell. Therefore, the out-of-plane polarization *P* is defined as

$$P=\frac{1}{V}\int_{n}^{n+\frac{C}{2}} z\rho(z)dz$$

where, *z* is the coordinate, *ρ* is the charge density, *V* represents the volume of half of the unit cell, *C* is the lattice constant of the cell in the *z*-axis direction, and *n* is the center of symmetry of the spatial inversion of the lattice structure.


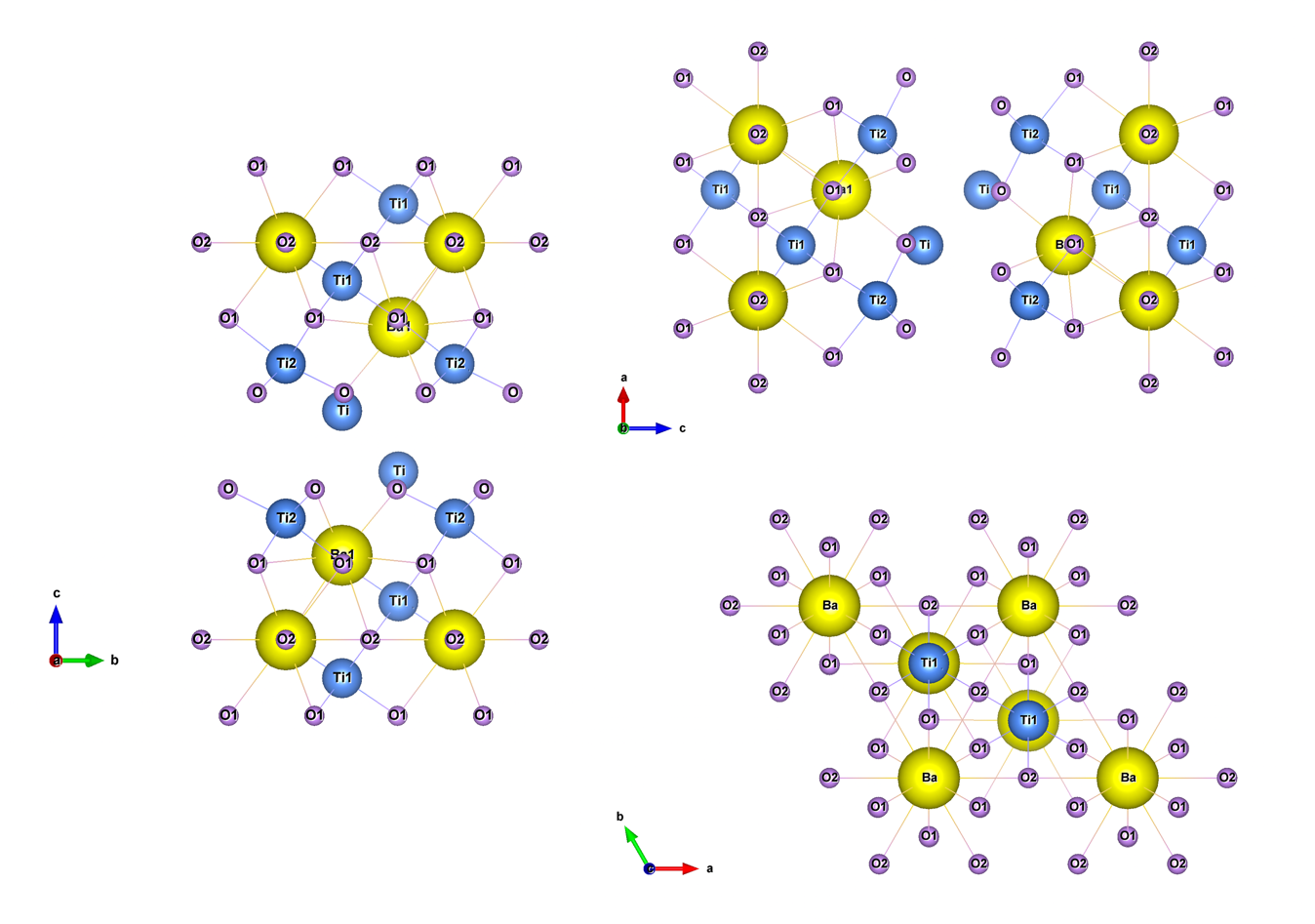


**Figure S1.** BT2 single unit cell along a, b and c axis with atomic labels.

The construction of the basic unit cell matrix originated from the peak fitting of the powder diffraction pattern, indicating that the phase is similar to the pre-existing structure Ba_3_La_2_Ti_2_Nb_2_O_15_ (mp-1228337_conventional standard cif file), belonging to the A_5_B_4_O_15_-type hexagonal perovskites. According to the elemental distribution ratio of BT_2_, as indicated by STEM EDS and XPS (Ba:Ti = 1:2), cations in A_5_B_4_O_15_ have been replaced to match the contents of BT2. After atomic replacement, the unit cell was used for Rietveld refinement in GSAS.

**Table S1.** Structural parameters of new BT2 from Rietveld refinement of synchrotron powder diffraction pattern.

| **No.** | **Elements** | **Label** | **x** | **y** | **z** | **Occ.** | **Site** | **Sym.** |
| --- | --- | --- | --- | --- | --- | --- | --- | --- |
| 1 | Ba | Ba | 0.00000 | 0.00000 | 0.00000 | 1.000 | 1a | -3m. |
| 2 | Ba | Ba1 | 0.66667 | 0.33333 | 0.21280 | 1.000 | 2d | 3m. |
| 3 | Ti | Ti | 0.66667 | 0.33333 | 0.57610 | 1.000 | 2d | 3m. |
| 4 | Ti | Ti1 | 0.66667 | 0.33333 | 0.90341 | 1.000 | 2d | 3m. |
| 5 | Ti | Ti2 | 0.00000 | 0.00000 | 0.30530 | 1.000 | 2c | 3m. |
| 6 | O | O | 0.34344 | 0.17172 | 0.37871 | 1.000 | 6i | . m. |
| 7 | O | O1 | 0.33846 | 0.16923 | 0.80863 | 1.000 | 6i | . m. |
| 8 | O | O2 | 0.50000 | 0.00000 | 0.00000 | 1.000 | 3e | .2/m. |

Abbreviations: Occ. (Occupancy) and Sym. (Symmetry).

**Figure S2.** STEM gallery of BT2 nanoparticles.


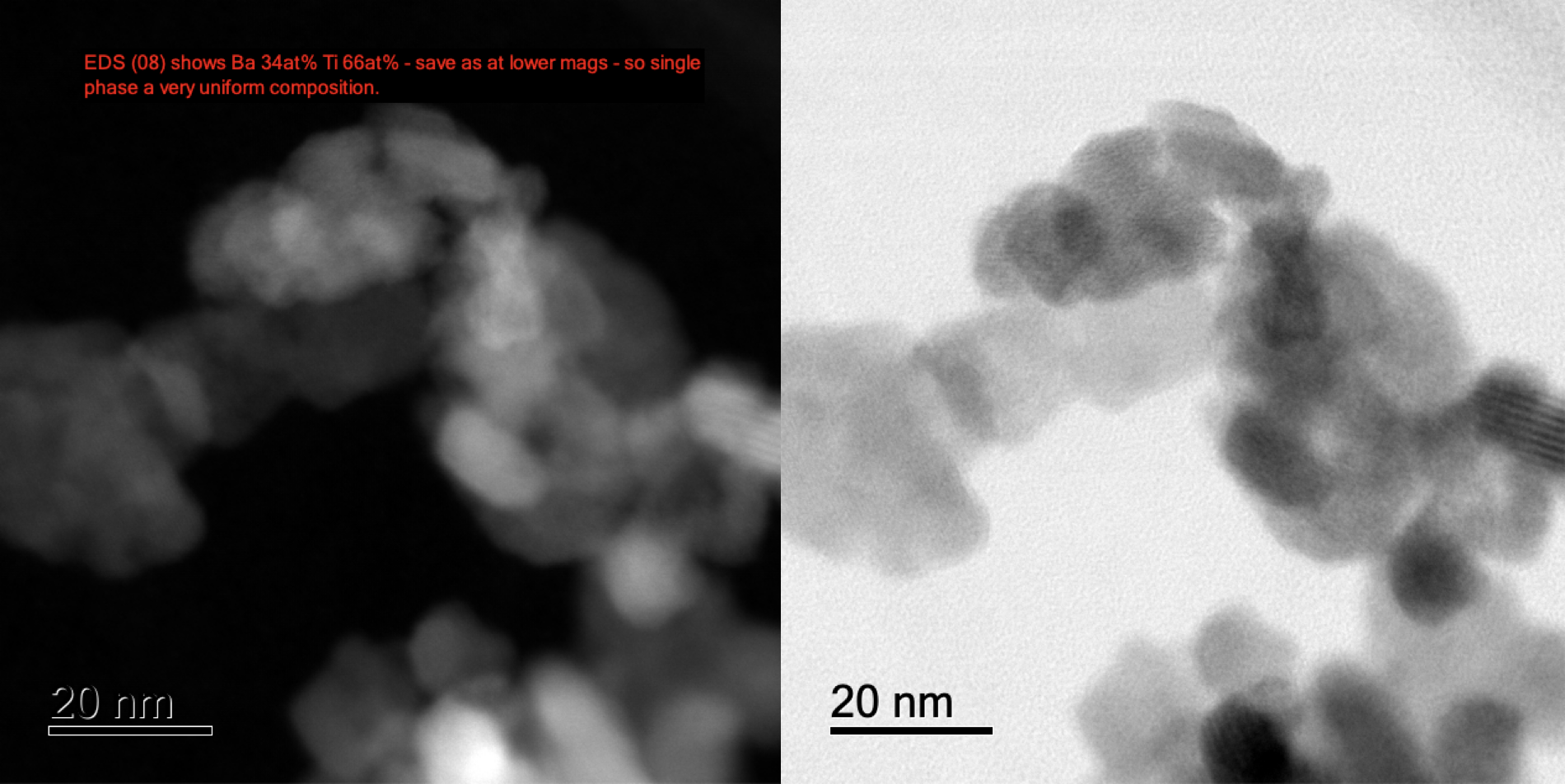


The illustration written in red was from the beam scientist to show the contents distribution of BT2.


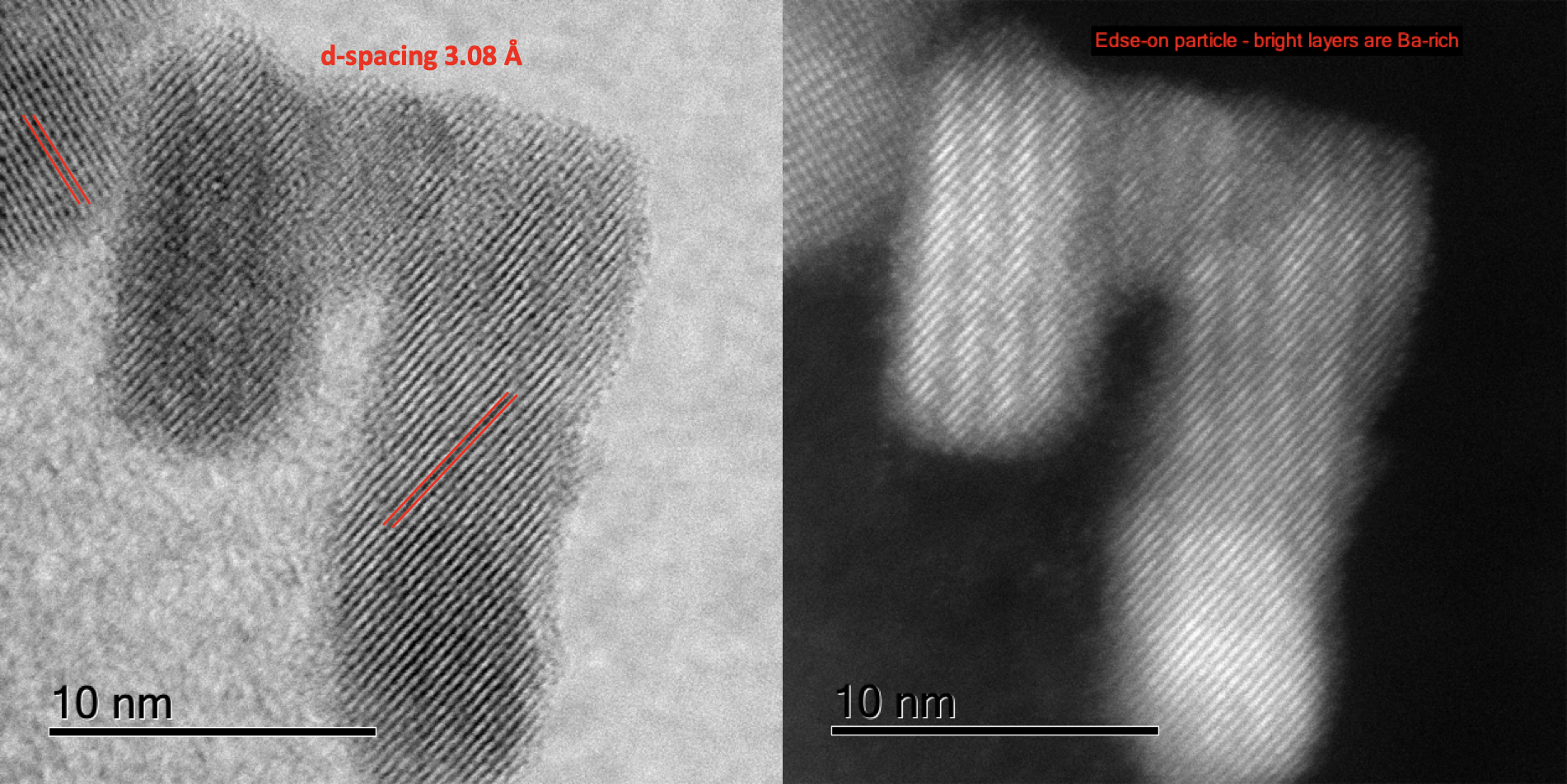


Here, we can see the measured d-spacing is 3.08 Å, and the corresponding crystalline plane can be found in Table S2.


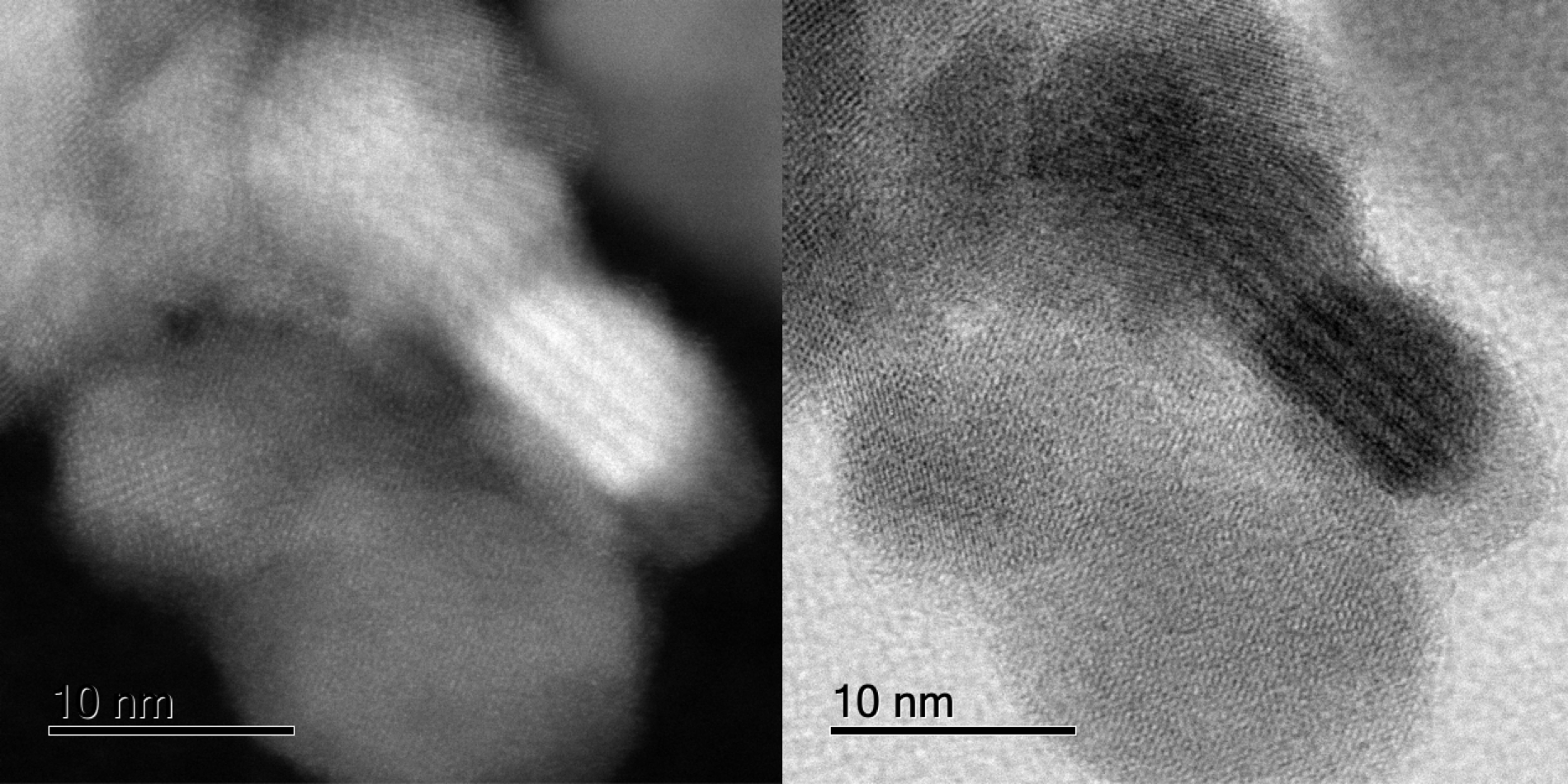


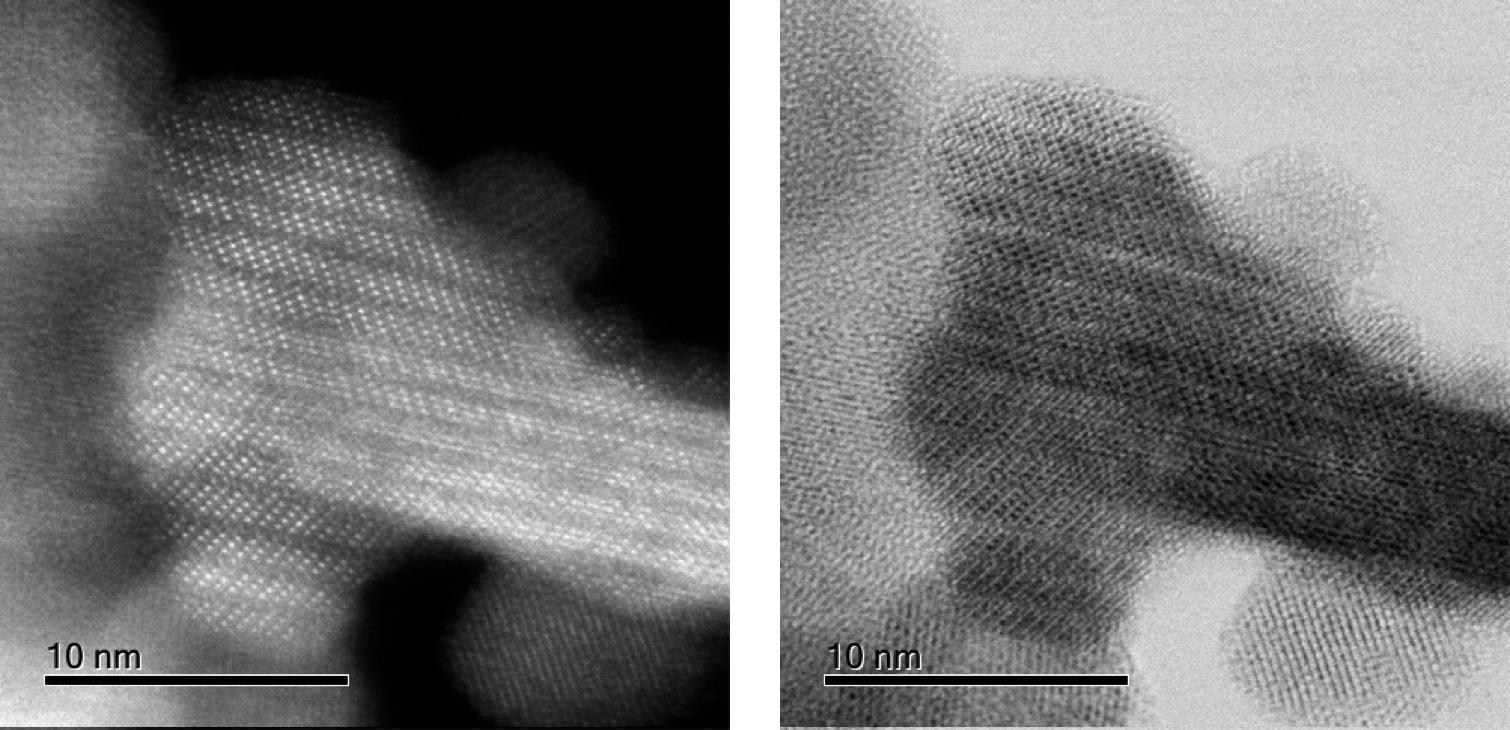


Above figures show the atomic distribution of the BT2, which is in consistent with the one shown in the main text.


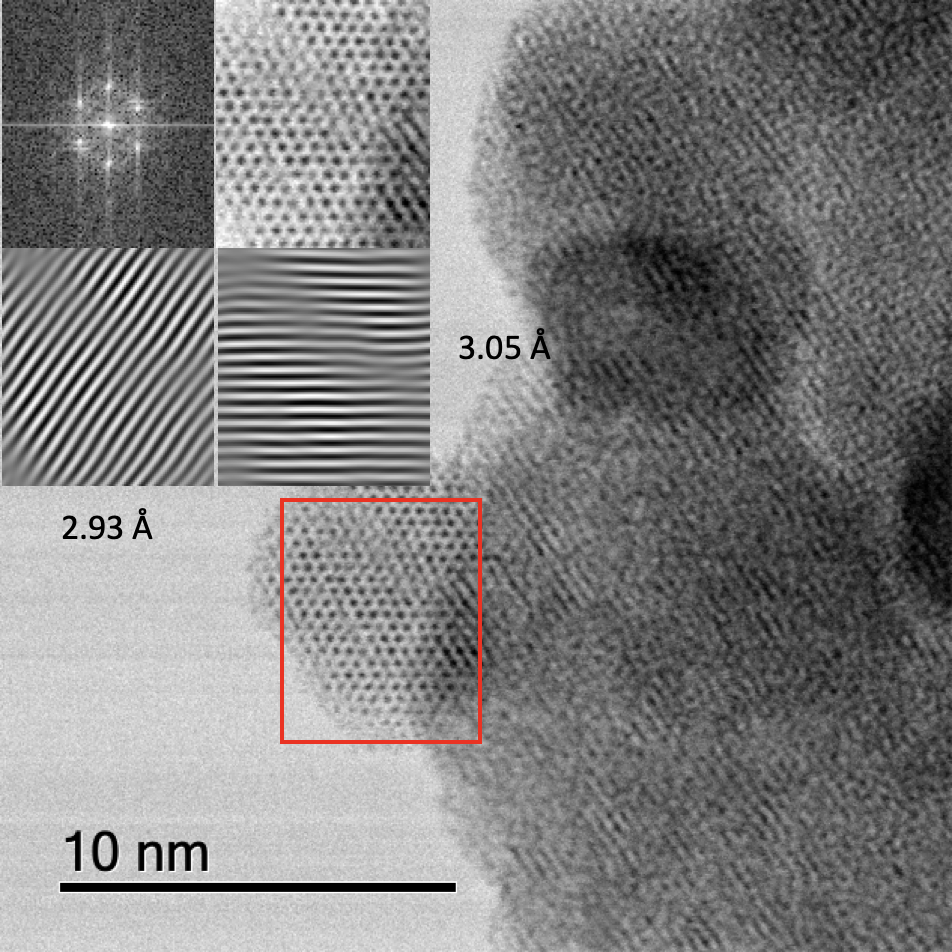


Insets of the above STEM image show the diffractogram achieved from reduced FFT from DigitalMicrograph, as well as the d-spacing measurement.


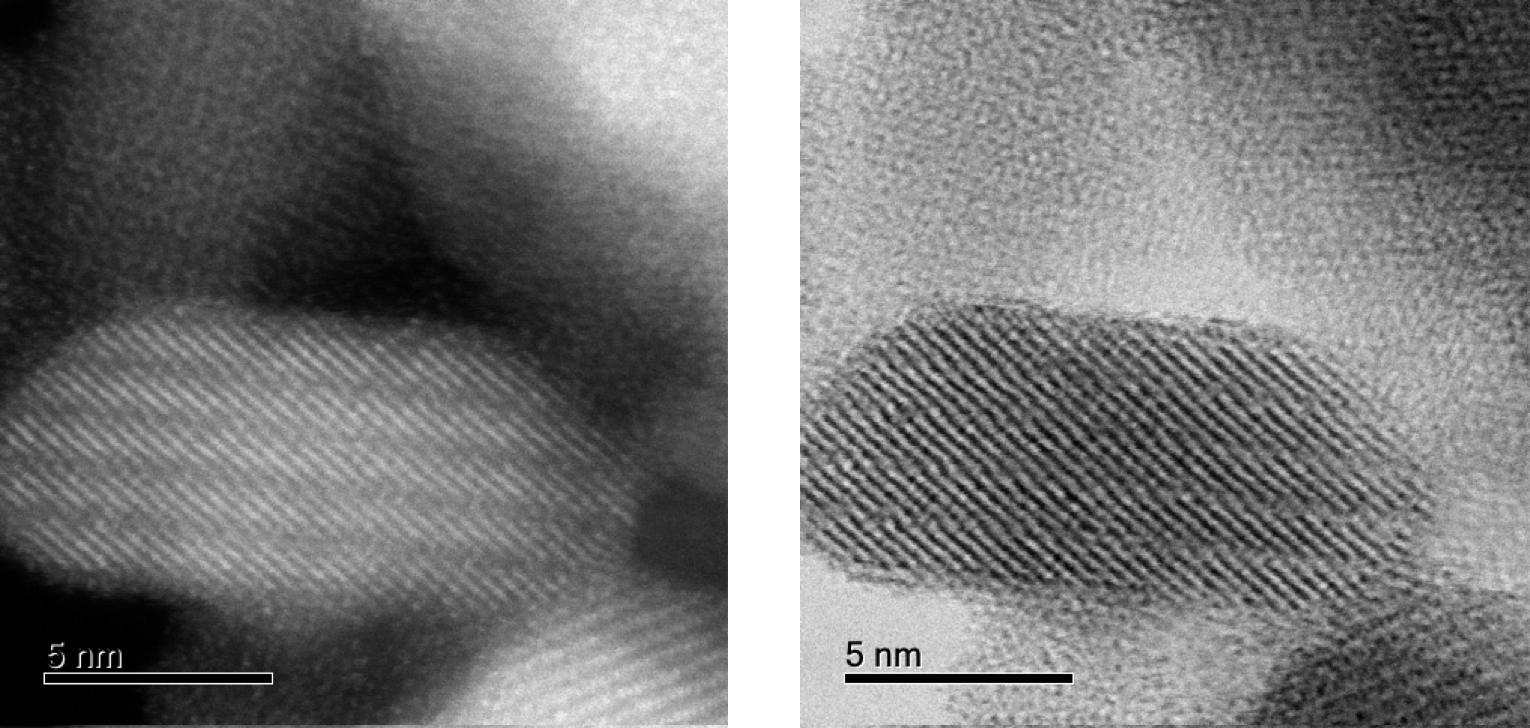


As shown above, the particle size is around 10 nm.

**Table S2.** Crystalline lattice plane details of BT2 derived from SPD refinement including the d-spacing shown in the STEM images.

| H | K | L | d | 2 theta |
| --- | --- | --- | --- | --- |
| 0 | 0 | 2 | 5.8245 | 5.8240 |
| 1 | -1 | 0 | 4.9496 | 6.8442 |
| 1 | 0 | 0 | 4.9497 | 6.8441 |
| 0 | 1 | 0 | 4.9493 | 6.8447 |
| 1 | -1 | -1 | **4.5555** | 7.4321 |
| 1 | -1 | 1 | **4.5554** | 7.4322 |
| 1 | 0 | 1 | **4.5554** | 7.4325 |
| 0 | 1 | -1 | **4.5552** | 7.4325 |
| 0 | 1 | 1 | **4.5551** | 7.4326 |
| 0 | 0 | 3 | 3.8830 | 8.7114 |
| 1 | -1 | -2 | 3.7717 | 8.9672 |
| 1 | -1 | 2 | 3.7716 | 8.9674 |
| 1 | 0 | -2 | 3.7718 | 8.9670 |
| 1 | 0 | 2 | 3.7716 | 8.9675 |
| 0 | 1 | -2 | 3.7716 | 8.9676 |
| 0 | 1 | 2 | 3.7715 | 8.9677 |
| 1 | -1 | -3 | **3.0551** | 11.0628 |
| 1 | -1 | 3 | **3.0550** | 11.0632 |
| 1 | 0 | -3 | **3.0552** | 11.0626 |
| 1 | 0 | 3 | **3.0550** | 11.0632 |
| 0 | 1 | -3 | **3.0550** | 11.0632 |
| 0 | 1 | 3 | **3.0549** | 11.0634 |
| 0 | 0 | 4 | **2.9122** | 11.6045 |
| 1 | -1 | -5 | 2.1080 | 24.0965 |
| 1 | -1 | 5 | 2.1079 | 24.0966 |
| 1 | 0 | -5 | 2.1080 | 24.0965 |
| 0 | 1 | -5 | 2.1079 | 24.0966 |
| 1 | 0 | 5 | 2.1079 | 24.0966 |
| 0 | 1 | 5 | **2.1079** | 24.0966 |
| 0 | 0 | 6 | **1.9415** | 17.4154 |


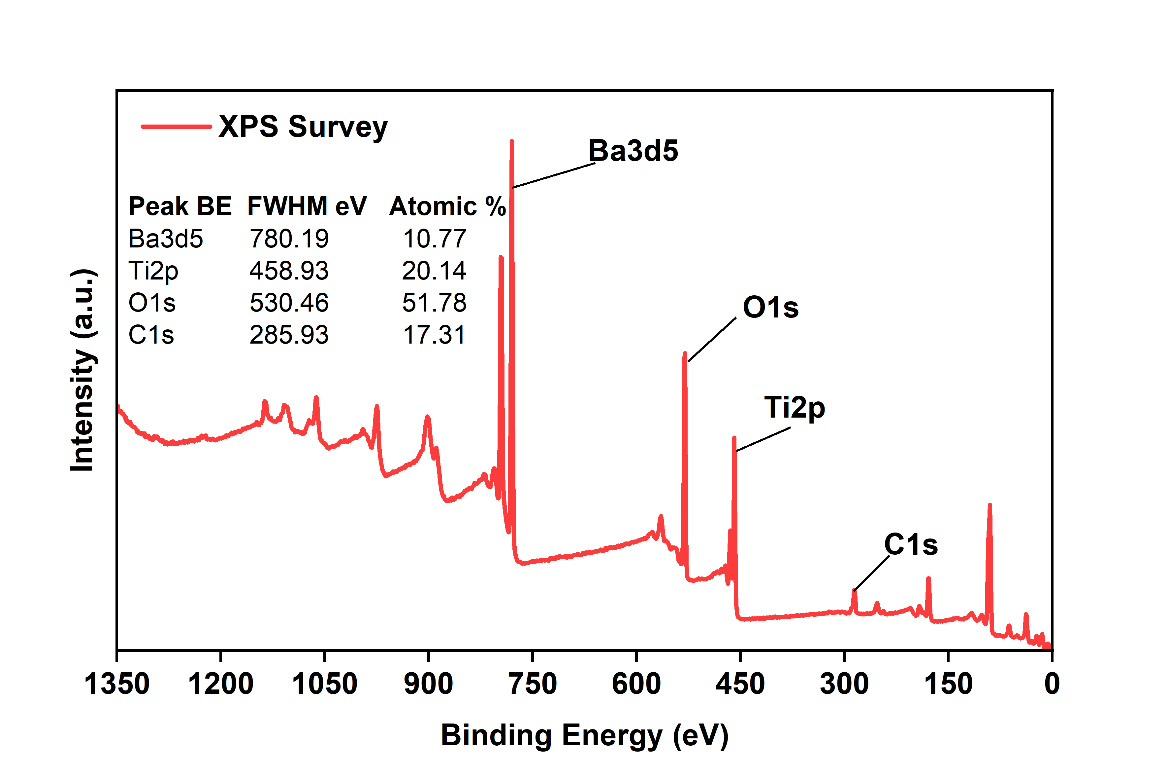


**Figure S3.** XPS survey of BT2 nanopowders with atomic contents distribution.


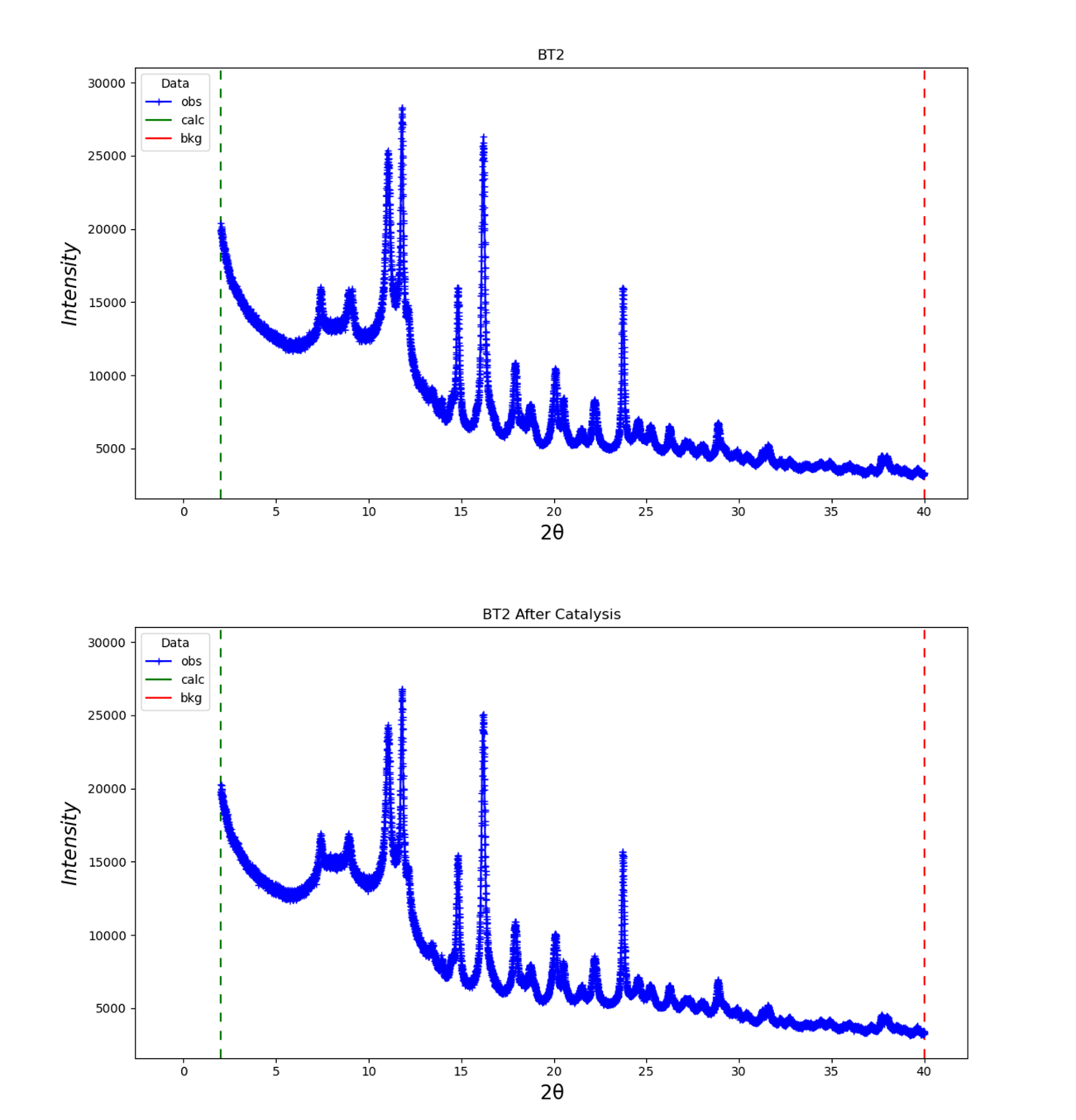


**Figure S4.** Synchrotron powder diffraction patterns of BT2 before and after hydrogen generation.

**Figure S5.** X-ray photoelectron spectroscopy (XPS) results of BT2 catalysts surface stability investigation. (a-c) BT2 XPS spectra before catalysis. (d-f) BT2 XPS spectra after hydrogen generation. (g-i) BT2 XPS spectra after organic RhB dye degradation.

**Figure S5(a-c)** shows the XPS spectra of the BT2 nanopowders before catalysis. The signals observed at 780 eV and 795 eV in **Figure S5a** represent the Ba 3 d_5/2_ and Ba 3 d_3/2_ states of barium oxide, and notably, they remain stable after catalysis, as illustrated in **Figure S5d and S5g**. For the observed signal O 1 s in **Figure S5b**, the signal at 530 eV represents the lattice oxygen in BT2, while the extended tail at around 532 eV is attributed to the surface moisture such as absorbed water molecules and surface hydroxyls.^[6]^ After hydrogen generation, the increase in the peak area of surface moistures in **Figure S5e** reveals a minor surface modification that attracted more polar molecules in water after prolonged vibration and immersion. This observation may provide insight into the earlier speculation regarding surface moisture absorption during immersion, as mentioned in the performance section. However, as shown in **Figure S5h**, after organic dye degradation, the O 1s peak of surface moisture at around 532 eV did not increase as significantly as the one observed during hydrogen generation. Instead, it presents a signal at around 531 eV, indicating organic adsorption during the catalytic process. Moreover, signals observed in **Figure S5f and S5i**  representing the Ti 2p_3/2_ and Ti 2p_1/2_ of Ti(IV) at 458 eV and 464 eV respectively, remain stable after catalysis compared to the Ti spectrum of original BT2 in **Figure S5c**.


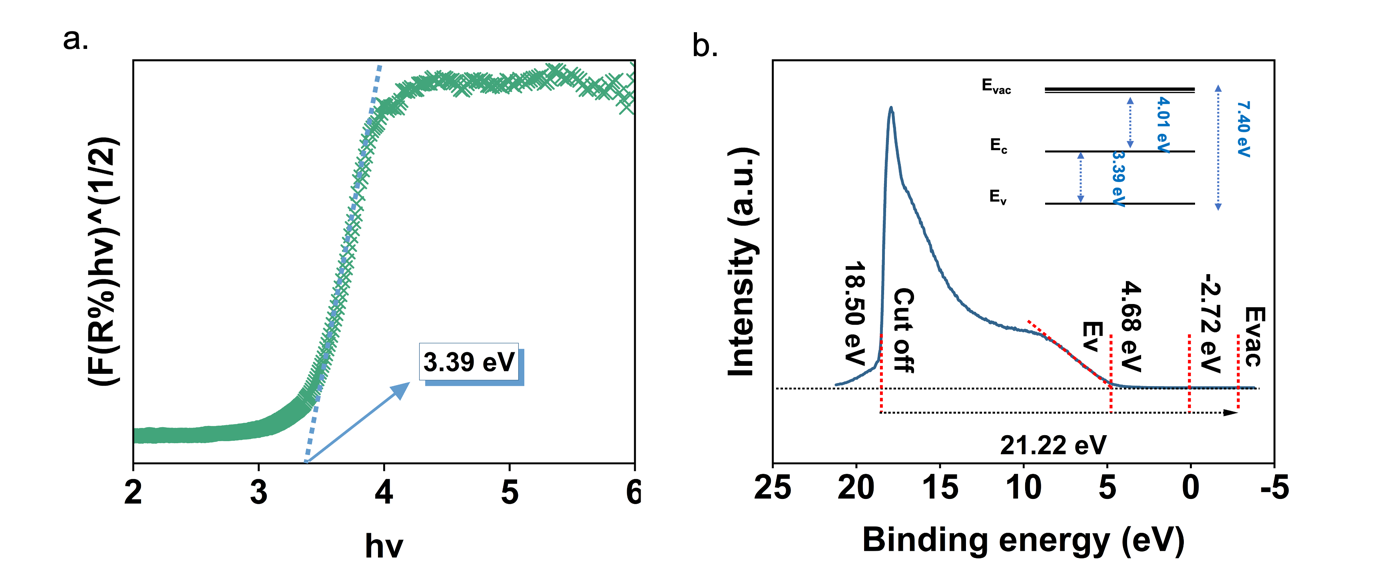


**Figure S6.** (a) A plot based on the Kubelka-Munk function transferred from diffused reflectance spectrum (DRS) by UV-Visible spectroscopy. (b) UPS spectrum of BT2 sample with band diagram estimation.

Energy band diagram of BT2 was estimated from DRS by UV-Visible spectroscopy and UPS. The band gap is around 3.39 eV as shown in Figure S5a. Valence band of BT2 was derived from UPS spectrum as demonstrated in Figure S5b, with estimated energy band diagram (vs. Evac) in the top left conner.

**Reference:**

[1] B. Ravel, M. Newville, *Journal of synchrotron radiation* **2005**, *12*, 537-541.

[2] a) W. Kohn, L. J. Sham, *Physical review* **1965**, *140*, A1133; b) P. Hohenberg, W. Kohn, *Physical review* **1964**, *136*, B864.

[3] a) G. Kresse, J. Hafner, *Physical review B* **1993**, *47*, 558; b) G. Kresse, J. Furthmüller, *Physical review B* **1996**, *54*, 11169.

[4] J. P. Perdew, K. Burke, M. Ernzerhof, *Physical review letters* **1996**, *77*, 3865.

[5] H. J. Monkhorst, J. D. Pack, *Physical review B* **1976**, *13*, 5188.

[6] Y. Du, T. Lu, X. Li, Y. Liu, W. Sun, S. Zhang, Z. Cheng, *Nano Energy* **2022**, *104*, 107919.
